# Compliance With a Risk-Factor-Based Guideline for the Prevention of Neonatal Group B Streptococcal Sepsis

**DOI:** 10.1155/S1064744997000604

**Published:** 1997

**Authors:** Maureen T. Fleming, Robert S. McDuffie, Kathy Russell, Susan Meikle

**Affiliations:** Department of Obstetrics and Gynecology St. Joseph Hospital and Kaiser Permanente 1835 Franklin Street Denver CO USA

## Abstract

*Objective:* The purpose of this study was to determine the compliance rate with a maternal risk-factor-based guideline for the prevention of neonatal group B streptococcal (GBS) sepsis.

*Methods:* In August 1994, a risk-factor-based guideline for selective intrapartum prophylaxis against neonatal GBS was adopted by a group model health maintenance organization. This guideline identified the following maternal risk factors for neonatal GBS sepsis: preterm delivery, rupture of membranes for >18 h, fever/chorioamnionitis, and history of a previous GBS-affected child. Patients with one or more risk factors were to receive intrapartum antibiotic prophylaxis consisting of either ampicillin, erythromycin, or clindamycin. We conducted a retrospective chart review to record risk factors and use of antibiotics. We hypothesized that >90% of patients with risk factors would receive intrapartum chemoprophylaxis.

*Results:* A total of 805 maternal charts were reviewed. Of these, 105 (13%) were candidates for intrapartum prophylaxis. We found an overall compliance rate of 65%. Compliance rates by risk factor were preterm delivery (51%), prolonged rupture of membranes (73%), fever/chorioamnionitis (87%), and previous affected child (100%).

*Conclusions:* Our results show unexpectedly low compliance rates with a risk-factor-based guideline for the prevention of neonatal GBS sepsis. Only 65% of women with any risk factor for neonatal GBS sepsis received intrapartum antibiotic prophylaxis appropriately. Educational efforts to improve compliance with a risk-factor-based guideline should specifically address mothers delivering at 34–36 weeks gestation and mothers with prolonged rupture of membranes.


Infectious Diseases in Obstetrics and Gynecology 5:345–348 (1997)
© 1998 Wiley-Liss, Inc.

### Compliance With a Risk-Factor-Based Guideline for the Prevention of Neonatal Group B Streptococcal Sepsis

**Maureen T. Fleming,* Robert S. McDuffie, Jr., Kathy Russell, and Susan Meikle**

*Department of Obstetrics and Gynecology, St. Joseph Hospital and Kaiser Permanente, Denver, CO*

### Abstract

*Objective:* The purpose of this study was to determine the compliance rate with a maternal risk-factor-based guideline for the prevention of neonatal group B streptococcal (GBS) sepsis.

*Methods:* In August 1994, a risk-factor-based guideline for selective intrapartum prophylaxis against neonatal GBS was adopted by a group model health maintenance organization. This guideline identified the following maternal risk factors for neonatal GBS sepsis: preterm delivery, rupture of membranes for >18 h, fever/chorioamnionitis, and history of a previous GBS-affected child. Patients with one or more risk factors were to receive intrapartum antibiotic prophylaxis consisting of either ampicillin, erythromycin, or clindamycin. We conducted a retrospective chart review to record risk factors and use of antibiotics. We hypothesized that >90% of patients with risk factors would receive intrapartum chemoprophylaxis.

*Results:* A total of 805 maternal charts were reviewed. Of these, 105 (13%) were candidates for intrapartum prophylaxis. We found an overall compliance rate of 65%. Compliance rates by risk factor were preterm delivery (51%), prolonged rupture of membranes (73%), fever/chorioamnionitis (87%), and previous affected child (100%).

*Conclusions:* Our results show unexpectedly low compliance rates with a risk-factor-based guideline for the prevention of neonatal GBS sepsis. Only 65% of women with any risk factor for neonatal GBS sepsis received intrapartum antibiotic prophylaxis appropriately. Educational efforts to improve compliance with a risk-factor-based guideline should specifically address mothers delivering at 34–36 weeks gestation and mothers with prolonged rupture of membranes. Infect. Dis. Obstet. Gynecol. 5:345–348, 1997. © 1998 Wiley-Liss, Inc.

Key words compliance; group B sepsis; maternal risk factors; neonatal sepsis

The best strategy to prevent early-onset neonatal group B streptococcal (GBS) sepsis is controversial. During the past 2 decades, investigators have proposed several options including 1) antepartum treatment of women known to be colonized with GBS, 2) intrapartum chemoprophylaxis to either culture-positive women or women with known risk factors, 3) early administration of penicillin to all neonates, and 4) universal immunization of all pregnant women. Clinical trials have demonstrated that intrapartum prophylaxis is effective for prevention of neonatal GBS sepsis.[1–7] Until mid-1996, consensus regarding the optimum protocol had not yet been reached between the Centers for Disease Control and Prevention (CDC), the American Academy of Pediatrics (AAP), and the American College of Obstetricians and Gynecologists (ACOG). The differing positions of these organizations reflect practical concerns regarding feasibility, efficacy, and cost-effectiveness.

*Correspondence to: Dr. Maureen T. Fleming, Department of Obstetrics and Gynecology, St. Joseph Hospital, 1835 Franklin Street, Denver, CO 80218-1191.

Received 18 March 1997

Accepted 29 August 1997

Clinical Study

Compliance limits the success of any protocol. Complex protocols may have a low compliance rate. Gibbs et al.[8] implemented a guideline in a university hospital setting in which they cultured all women for GBS at 26–28 weeks gestation and treated intrapartum those women who were GBS positive and had risk factors. Over 2 years, the compliance rate was 80%. There were 5 cases of neonatal GBS sepsis which were associated with either protocol violations, protocol failures, or both. We implemented a guideline based solely on maternal risk factors in a health maintenance organization (HMO) setting. We hypothesized that this simpler protocol would have a 90% compliance rate.

### METHODS

In August 1994, the Department of Obstetrics and Gynecology at Kaiser Permanente, a group model HMO in Denver, CO, adopted a guideline for the prevention of early-onset neonatal GBS sepsis. This guideline was adapted from ACOG guidelines[9] and identified the following as maternal risk factors for neonatal GBS sepsis: preterm delivery (<37 weeks), rupture of membranes >18 h, maternal fever in labor (>38°C) accompanied by clinical signs of intraamniotic infection, and previous child affected by GBS. Kaiser Permanente patients deliver at one of two area hospitals and over 90% deliver at St. Joseph Hospital in Denver, CO. Care of these patients is provided by Kaiser Permanente attending obstetricians and by St. Joseph Hospital obstetrical and family practice house staff. All patients with one or more risk factors were to receive intrapartum antibiotic prophylaxis consisting of ampicillin 2 g intravenously every 6 h until delivery or, if allergic to penicillin, erythromycin 500 mg intravenously every 6 h or clindamycin 900 mg intravenously every 8 h. Cultures for GBS were not collected unless the patient presented with preterm premature rupture of membranes (PPROM). All women at their initial obstetrical visit underwent urinary screening with a dipstick. Positive screening was defined as the presence of nitrites or leukocytes on dipstick, or >10 WBC/hpf or 3+ bacteria on microscopic examination. Patients who had positive screening had urinary cultures performed. Women with urinary cultures positive for GBS were treated and were not included in this guideline for prophylaxis. During this time period, patients with idiopathic preterm labor unlikely to deliver and patients with PPROM not in labor were not routinely treated with antibiotics.

Implementation of this risk-factor-based protocol was assisted with educational conferences provided to all attending obstetricians, house staff, and nurses involved with direct patient care of laboring patients. Copies of the guidelines were posted in the labor and delivery suites and provided to all involved personnel. Standing orders included notification of physicians by the nursing staff when women were <37 weeks gestational age or had rupture of membranes for >18 h.

We retrospectively reviewed charts of all Kaiser Permanente patients delivering at St. Joseph Hospital between January 1, 1995, and March 31, 1995. We identified these patients by an existing perinatal data base. Since we hypothesized that compliance would be 90%, a 3 month sample of delivering mothers was determined to have 80% power to detect a difference in compliance of 10%. We excluded patients with deliveries before 24 weeks of gestation or with a fetal demise at any gestational age. We reviewed charts, using a standard form, for gravidity, parity, gestational age at delivery, presence of risk factors, and administration of intravenous antibiotics. Documentation of antibiotic administration was obtained from the nursing record and not from physician orders. Charts with missing values or those showing non-compliance with the protocol, i.e., no antibiotics given to a patient with a risk factor, were re-audited by two investigators. We compared the overall compliance rate with the anticipated rate by the chi-square test and considered $P < 0.05$ to be significant.

### RESULTS

A total of 805 maternal charts were reviewed. We could not locate 4 charts of patients delivering during this time period. Of these patients, 105 (13%) were candidates for intrapartum prophylaxis because of maternal risk factors. Sixty-eight of these patients received antibiotic prophylaxis for an overall compliance rate of 65% ($P < 0.001$, compared to predicted value). Compliance rates by risk factor are listed in Table 1.

We observed a preterm delivery rate of 6% (51 of 805). For women with preterm delivery, the compliance rate was 51% (26 of 51). This group had the lowest compliance rate in this study. Of the patients with preterm delivery who failed to re-

TABLE 1. Maternal risk factors for neonatal GBS sepsis by prevalence and compliance rate[a]

| Risk factor | Risk factor prevalence | Compliance rate |
|---|---|---|
| Preterm delivery | 51/805 (6%) | 26/51 (51%) |
| Rupture of membranes >18 h | 37/805 (5%) | 27/37 (73%) |
| Fever/chorioamnionitis | 30/805 (4%) | 26/30 (87%) |
| Previous affected child | 3/805 (0.4%) | 3/3 (100%) |
| Any risk factor | 105/805 (13%) | 68/105 (65%) |

[a]Compliance is defined as administration of appropriate antibiotics after identification of appropriate risk factors.

ceive intrapartum antibiotic prophylaxis, all were between 34 and 36 weeks of gestation.

We found prolonged rupture of membranes in 5% (37 of 805) of women. For these, the compliance rate was 73% (27 or 37). Of the non-compliant cases, 6 of 10 (60%) had a duration of rupture of membranes between 18 and 19 h and 9 of 10 (90%) had a duration of rupture of ≤24 h. There were 3 patients with a previous child affected by GBS; all of these received intrapartum antibiotics. Of 5 women with a positive urinary or genital GBS culture, 4 received intrapartum prophylaxis in the absence of other specified risk factors.

Two neonates during the study period were treated after birth for blood culture positive GBS sepsis. Neither mother received intrapartum antibiotic therapy nor had any risk factor. One mother underwent induction at 40 weeks of gestation for a low amniotic fluid index. She received two prostaglandin suppositories and oxytocin and delivered within 14 h after placement of the first suppository. Membranes were ruptured 3 h before delivery, and the mother was afebrile during her labor. The neonate was admitted to the neonatal intensive care unit (NICU) for hypoglycemia and blood cultures were subsequently positive for GBS. The second patient labored spontaneously at 40 weeks of gestation. Membranes were ruptured for 8 h prior to delivery and the mother was afebrile during her labor. After a forceps delivery, the baby was taken to the NICU for presumptive meconium aspiration. Both babies were discharged within 14 days of delivery, received home intravenous antibiotic therapy, and recovered without sequelae.

### DISCUSSION

We found that compliance with this risk-factor-based guideline for the prevention of neonatal GBS sepsis was lower than predicted (65% vs. 90%). This lower rate was due largely to non-compliance in two risk factor categories: delivery before 37 weeks gestation and rupture of membranes longer than 18 h. For the former, all cases of non-compliance occurred between 34 and 36 weeks of gestation. For prolonged rupture of membranes, 90% (9 of 10) of guideline violations occurred when time from rupture to delivery was between 18 and 24 h.

We offer several explanations for the lower compliance with this guideline in these two risk factor categories. First, tocolysis is controversial at 36–37 weeks of gestation and these mothers may not have been labeled "preterm." Also, several patients in this risk factor category were induced for preeclampsia, and the clinicians involved with their care may have focused more on hypertension than prematurity, thus failing to identify these women as candidates for intrapartum prophylaxis. Second, some women experienced a rapid labor, and there may not have been time to administer chemoprophylaxis. Historically, clinicians have been taught to have increased suspicion for chorioamnionitis starting at 24 h after rupture of membranes. Starting antibiotic therapy at 18 h may represent a change in practice and patients at 18 h may not have been identified as being at risk. An additional factor was the number of physicians involved. Nearly 40 physicians rotated in attendance at these births. Finally, even the use of "standard" written orders to notify physicians in situations when GBS prophylaxis is indicated did not prevent protocol violations. This may reflect the use of verbal orders on labor and delivery. Educational efforts aimed at improving identification of appropriate candidates, particularly those with gestational age of 34–36 weeks and rupture of membranes between 18 and 24 h, might improve compliance considerably.

In programs that culture all pregnant women for GBS and treat those women who test positive, the rate of intrapartum chemoprophylaxis may be as high as 40%.[10] There is concern that chemoprophylaxis of a high percentage of women in labor will result in the selection of antibiotic-resistant bacteria strains. In this risk-factor-based guideline, 13% (105/805) of the population were candidates for intrapartum chemoprophylaxis. This is less than the 18% predicted by Rouse et al.,[11] who used a decision analysis to compare rates of chemoprophylaxis using culture vs. risk-factor-based guidelines.

The CDC recently published guidelines that offer a choice between risk-factor-based and mixed risk-factor/culture-based guidelines for intrapartum chemoprophylaxis.[12] The decision on which guideline to use should be based not only on rates of GBS carriage, incidence of neonatal sepsis, and risk factors, but also on practical concerns including compliance. Although this risk-factor-based guideline did not eliminate neonatal GBS sepsis, it did address those infants most likely to experience the greatest morbidity and mortality while prophylaxing a reasonable percentage of women (13%). Successful implementation of guidelines requires education, provider feedback, and evaluation of the contribution to health care. Educational efforts which target the risk factors of preterm delivery and premature rupture of membranes may improve compliance with this guideline.

### REFERENCES

1. Yow MD, Mason EO, Leeds LJ, Thompson PK, Clark DJ, Gardner SE: Ampicillin prevents intrapartum transmission of group B streptococcus. JAMA 241:1245–1247, 1979.
2. Easmon CSF, Hastings MJG, Deeley J, Bloxham B, Rivers RPS, Marwood R: The effect of intrapartum chemoprophylaxis on the vertical transmission of group B streptococci. Br J Obstet Gynaecol 90:633–635, 1983.
3. Boyer KM, Gadzala CA, Kelly PD, Gotoff SP: Selective intrapartum chemoprophylaxis of neonatal group B streptococcal early-onset disease. III. Interruption of mother-to-infant transmission. J Infect Dis 148:810–816, 1983.
4. Matorras R, Garcia-Perea A, Omenaca F, Diez-Enciso M, Madero R, Usnadizaga JA: Intrapartum chemoprophylaxis of early-onset group B streptococcal disease. Eur J Obstet Gynecol Reprod Biol 40:57–62, 1991.
5. Lim DV, Morales WJ, Walsh AF, Kazanis D: Reduction of morbidity and mortality rates for neonatal group B streptococcal disease through early diagnosis and chemoprophylaxis. J Clin Microbiol 23:489–492, 1986.
6. Allardice JG, Baskett TF, Seshia MMK, Bowman N, Malazdrewicz R: Perinatal group B streptococcal colonization and infection. Am J Obstet Gynecol 142:617–620, 1982.
7. Garland SM, Fliegner JR: Group B streptococcus and neonatal infections: The case for intrapartum chemoprophylaxis. Aust NZ J Obstet Gynaecol 31:119–122, 1991.
8. Gibbs RS, McDuffie RS, McNabb F, Fryer GE, Miyoshi T, Merenstein G: Neonatal group B streptococcal sepsis during 2 years of a universal screening program. Obstet Gynecol 84:496–500, 1994.
9. American College of Obstetricians and Gynecologists: Group B streptococcal infections in pregnancy. ACOG Technical Bulletin No. 170. Washington, DC: American College of Obstetricians and Gynecologists, 1992.
10. Anthony BF, Eisenstadt R, Carter J, Kim KS, Habel CJ: Genital and intestinal carriage of group B streptococci during pregnancy. J Infect Dis 143:761–766, 1981.
11. Rouse DJ, Goldenberg RL, Cliver SP, Cutter GR, Mennemeyer ST, Faragason CA: Strategies for the prevention of early-onset neonatal group B streptococcal sepsis: A decision analysis. Obstet Gynecol 83:483–494, 1994.
12. Prevention of perinatal group B streptococcal disease: A public health perspective. MMWR 45(RR-7):1–24, 1996.
